# Perspectives on child diarrhoea management and health service use among ethnic minority caregivers in Vietnam

**DOI:** 10.1186/1471-2458-11-690

**Published:** 2011-09-06

**Authors:** Thilde Rheinländer, Helle Samuelsen, Anders Dalsgaard, Flemming Konradsen

**Affiliations:** 1 Department of International Health, Immunology and Microbiology, University of Copenhagen, Denmark, Øster Farimagsgade 5, 1014 Copenhagen, Denmark; 2 Department of Anthropology, University of Copenhagen, Øster Farimagsgade 5, 1014, Copenhagen, Denmark, Denmark; 3 Department of Veterinary Disease Biology, University of Copenhagen, Denmark, Stigböjlen 4, 1870 Frederiksberg C, Denmark; 4 Department of International Health, Immunology and Microbiology, University of Copenhagen, Denmark, Øster Farimagsgade 5, 1014 Copenhagen, Denmark

## Abstract

**Background:**

In Vietnam, primary government health services are now accessible for the whole population including ethnic minority groups (EMGs) living in rural and mountainous areas. However, little is known about EMGs' own perspectives on illness treatment and use of health services. This study investigates treatment seeking strategies for child diarrhoea among ethnic minority caregivers in Northern Vietnam in order to suggest improvements to health services for EMGs and other vulnerable groups.

**Methods:**

The study obtained qualitative data from eight months of field work among four EMGs in lowland and highland villages in the Northern Lao Cai province. Triangulation of methods included in-depth interviews with 43 caregivers of pre-school children (six years and below) who had a case of diarrhoea during the past month, three focus group discussions (FGDs) with men, and two weeks of observations at two Communal Health Stations (CHGs). Data was content-analyzed by ordering data into empirically and theoretically inspired themes and sub-categories assisted by the software NVivo8.

**Results:**

This study identified several obstacles for EMG caregivers seeking health services, including: gender roles, long travelling distances for highland villagers, concerns about the indirect costs of treatment and a reluctance to use government health facilities due to feelings of being treated disrespectfully by health staff. However, ethnic minority caregivers all recognized the danger signs of child diarrhoea and actively sought simultaneous treatment in different health care systems and home-based care. Treatments were selected by matching the perceived cause and severity of the disease with the 'compatibility' of different treatments to the child.

**Conclusions:**

In order to improve EMGs' use of government health services it is necessary to improve the communication skills of health staff and to acknowledge both EMGs' explanatory disease models and the significant socio-economic constraints they experience. Broader health promotion programs should address the significant gender roles preventing highland mothers from seeking health services and include family elders and fathers in future health promotion programs. Encouraging existing child health care practices, including continued breastfeeding during illness and the use of home-made rehydration solutions, also present important opportunities for future child health promotion.

## Background

### The government health system in Vietnam

Coverage of government primary health care (PHC) facilities has greatly improved in the past two decades in Vietnam. By the end of 2008, 99% of all communes (the local administrative unit in Vietnam) in the country had a communal health station (CHS), including the northern mountainous communes inhabited by ethnic minorities [1]. Communal health stations provide primary health care including free immunization for children, treatment for minor diseases and common drugs (including treatment of child diarrhoea and respiratory infections), assistance during deliveries and take part in health promotion activities in the communes including mother and child health education, and hygiene and sanitation education. This study was conducted in the Northern mountainous regions of Vietnam where 32% of CHSs having doctors and 86% having midwives or assistant doctors specialized in paediatric and obstetric care [2]. CHSs are small health stations situated in each commune, which refer seriously ill patients to larger inter-communal clinics and district hospitals, which provide facilities for surgeries and in-patient treatment as well as more diagnostic services (e.g. simple lab-tests etc.).

Most (97%) villages in the Northern regions of Vietnam also have active Village Health Worker (VHWs) [2], who form a crucial part of the PHC system. They are laymen with short-course training in health and human diseases, typically three months of training plus occasional upgrade courses. They work part-time and have five official tasks; perform health education, disease prevention including hygiene promotion, mother and child health care including advocacy of family planning, first aid and disease treatment, and to participate in health programs with authorities and CHSs (e.g. inform caregivers about child immunization and give education to caregivers about child nutrition, care and hygiene) [3]. Private health providers are also a crucial part of the wider health system in Vietnam including a large numbers of traditional medical and herbal experts, spiritualists, and private bio-medical practitioners [4]. It has also become normal practice among Vietnamese to purchase and self-medicate with over-the-counter drugs, many sold without prescriptions, from the large number of private drugstores and pharmacies [5-7].

### The health situation of ethnic minority children

Improving child health indicators is a major priority for the Vietnamese government, who have initiated several child health programs and policies under the National Target Programs on Health since the early 1990'ties. Prevention of communicable diseases, improving coverage of the expanded child immunization program and improving child nutrition status are some of the main child health objectives. These programs all aim at providing basic child health services and promotion to all parts of the country. To ensure affordability and increase coverage of services, the National Health Care Fund for the Poor was established in 2002 to provide insurance for ethnic minorities and poor communities, making all treatment and drugs at CHSs and district hospitals free of charge for those insured [8]. However, child health inequalities still exist between rural and urban, and between majority and minority population groups.

Diarrhoeal diseases are the number two leading cause of morbidity and is endemic in the northern mountainous regions of the country inhabited mainly by EMGs [9], as are parasitic infections of children [10]. It was recently estimated that 12% of children in a mountainous rural district of Vietnam had experienced diarrhoea within two weeks [11]. Children living in the northern and central highlands also experience higher risks of malnutrition compared with children living in urban and rural lowland [11,12]. A recent analysis of official statistics also highlights that far more ethnic minorities and children from the northern regions live in poverty, have poor shelter and limited coverage of water, sanitation and immunization compared with urban regions and Kinh children [13].

### Caregivers' use of health care services and drugs for child illness management

In principle, all caregivers in Vietnam have access to a well-established and free PHC system for children. And several studies have shown that self-medication of child illnesses is also common in urban as well as rural Vietnam [14] and that antibiotics are commonly prescribed, and overprescribed, to children [14-16]. One study in a Kinh populated rural district found that only 54% of caregivers used antibiotics and 36% used analgesic and antipyretics to treat the child's diarrhoea, while only 9.7% gave the child ORS [15]. Compared with the majority of Vietnamese (Kinh) parents, quantitative studies have found that ethnic minority caregivers are even less likely to give ORS and seek treatment at CHSs when children have diarrhoea [17,18]. One of these studies also found that ethnic minority caregivers perceived diarrhoea episodes as less severe compared with Kinh caregivers [18]. Indirect costs of treatment, user fees and long distances to health facilities have also been mentioned as important factors that hinder the use of health facilities among poor and the EMGs living in highland areas [19,20].

However, the perspectives of Vietnamese ethnic minority caregivers on child illness management and treatment preferences and perceptions are not well described in the literature. This study explores treatment seeking strategies among ethnic minority caregivers of children with diarrhoea in Northern Vietnam, in order to suggest improved health services for EMGs and other vulnerable groups in Vietnam.

## Methods

### Study area, population and health services

Six months of fieldwork was conducted in two adjacent rural communes in the Northern Province of Lao Cai in Vietnam in 2008, investigating the general living and working conditions, perceptions and practices of health, hygiene and sanitation among ethnic minorities [21]. For the current study, and building on previous findings, two additional months of fieldwork was conducted in 2009 focusing on caregivers' management and perceptions of child diarrhoea.

The total population of the two communes was 10,000 with 85% being ethnic minorities. Fieldwork was conducted among the four most populated minority groups in the area; Giáy, Tày, Xa Phó (living in lowland villages) and Red Dao (highland people). About 40% of households were below the government poverty line of 2006 - 2010 for rural areas (defined by a monthly income below 200,000 Vietnamese Dong per person, approximating 10.5 USD), with a higher concentration of poor households among the Xa Phó (living on the edge of the forested hills) and the Red Dao in the highland [22]. All ethnic minorities depend on subsistence rice farming, animal husbandry and forestry with all adults doing hard physical fieldwork daily. All four ethnic groups have traditionally lived in multigenerational households and are all patrilineal, with men being the heads of households and owners of all property, and patrilocal with daughters moving to their groom's families when married.

Further details on the study area and the minority groups have been reported earlier [21].

Two CHSs, one also hosting the Preventive Medicine Office in charge of conducting preventive activities in both communes, were located in the lowland part of each commune. Both CHSs were run by assistant doctors and had nurses, pharmacists and nurse midwives employed. Both CHSs had a selection of common drugs and remedies available including ORS, antibiotics, other anti-diarrhoeals and allopathic medicines. Village Health Workers had been appointed by the CHSs for almost all of the 39 villages in the communes. Government district and private hospitals and drug stores were located in the district town 3-5 kilometres away from the clinics. No private bio-medical practitioners, clinics or drug stores were found in the two communes. But several spiritualists (Thày Mo's) were identified as important health providers in highland villages, performing fortune telling and warding rituals to strengthen health. The lowland villages (Tày and Giáy) were located closest and within five kilometres to the CHSs. The Xa Phó villages were located within eight kilometres, while highland villages were scattered and located on steep dirt roads up in the mountains 10-15 kilometres away from the CHS. No public transportation exists and people walk, bike or drive motorbikes to the CHSs and district towns.

### Data Collection

The main source of data was 43 semi-structured in-depth interviews with caregivers of pre-school children (six years and below) who had experienced a case of diarrhoea in the past month. In addition, two weeks of observations of patient-staff interactions were performed at the two CHSs and three focus group discussions (FGDs) were conducted with men. Data from participant observations in four different study communities conducted in 2008 was included as general background information of the living conditions in highland and lowland settings (methodology has been described in details elsewhere [23]).

Information about diarrhoea cases in 10 lowland and two highland villages (Table 1) of the four ethnic groups were collected by asking caregivers if their child had experienced an incidence of loose or watery stools at least three times per 24 hours during the past month, using the diarrhoea definition of WHO [24]. Several sampling methods were used to ensure that cases included mild and severe cases, and cases treated at home and by various health providers. These included two weeks of observations at the two CHSs (eight cases identified), a project community diarrhoea survey covering both communes (seven cases), by references from VHW's (nine cases), and house-to-house visits by the first author and assistants in villages (19 cases).

**Table 1:** Characteristics of respondents

*Caregiver Interviews*	*Tày*	Giáy	*Xa Phó*	*Red Dao*	*Total*
Mothers	10	9	9	11	39
Fathers	-	-	-	2	2
Grandfather or mother	1	4	1	6	12
Age (range)	24-55	24-70	22-50	19-50	

***Focus group Discussions***					
Fathers	6	Not	8	3	17
Grandfathers	2	Conducted	0	5	7
Age (range)	25 - 61	24-70	25 - 43	24 - 50	

Interviews included questions on perceived causes of diarrhoea, decision making about treatment, treatment choices, and use of health services (See additional file 1: Caregiver Interview Guide). The main informant was the adult caregiver who had attended to the child during sickness, but during seven of the interviews more than one caregiver was present and answering questions for their children. Attempts were made to avoid having grandparents in the same interviews as mothers to allow mothers to talk openly about family decision making. To gain in-sight into influences on treatment seeking and choices of fathers and grandfathers, three FGD's were conducted among men from two lowland villages (Tày and Xa Phó groups) and a highland village (Red Dao). Characteristics of participants are shown in Table 1.

Interviews and FGDs were conducted in Vietnamese by two Vietnamese assistants with the first author being present during half of the interviews and all of the FGDs to ask elaborating and clarifying questions. Young local women assisted with translation during interviews in the highland and Xa Phó villages if the interviewees did not speak Vietnamese.

Caregivers with sick children encountered at the CHSs and in households were not interviewed, but asked if they were willing to be interviewed at a later time once the child had recovered. Two caregivers with sick children met at the CHS declined to be contacted again. One caregiver with a sick child undergoing treatment was met in a Xa Phó village and interviewed at a later stage. All interviews were audio recorded with informed verbal consent and promised full anonymity and confidentiality. Recordings were transcribed, cross-checked and translated ad verbatim into English by the two assistants. Data was coded for thematic content analysis [25] by the first author using pre-set and emerging empiric and theoretical categories by the NVivo 8 software program [26].

The study was approved by the National Institute of Hygiene and Epidemiology in Hanoi, The Department of Health in Lao Cai province, and Peoples' Committees (government authorities) in the two study communes.

## Results

Two ethnographic descriptions of two diarrhoea cases drawing on observations and interview data from various sources, illustrate the identified similarities and differences in perceptions of disease and health choices in a highland and lowland setting (Tables 2 and 3). These cases serve as a point of departure for the analysis and discussion of the underlying logic for treatment seeking in ethnic minority families.

**Table 2:** A Highland case of child diarrhoea management

*Discovering the disease* One evening when a 23-year old mother returns from field work in the hills, the children who have been attending her 12-months old baby say that the child has a 'hard belly' and hasn't defecated during the day. During the night the child defecates liquid and greenish stools thrice, cries, and develops a bellyache and slight fever. The mother describes the child as very sleepy, weak, and not wanting to eat anything except for taking breast milk.
***Treatment in the home*** The diarrhoea goes on for three days. During this period the parents-in-law allow the mother to remain home to attend to the child and cook for the family. When asked about the cause of the diarrhoea the mother is uncertain: "*Maybe because of the weather - but I don't know [...] I thought it was maybe because the child ate something unsuitable, drank dirty water or played dirtily"*. She explains 'unsuitable foods' as something which *'couldn't be digested' *by the child, including sweet milk or toxic forest fruits. For the first two days the mother carries the child around, all the while breastfeeding and urging the child to drink boiled water and eat some rice in the hope that the disease will cease. On the third day the parents-in-law decide to call the village 'thầy mo' (spiritualist). He explains how to cure diarrhoea *"First we have to do 'bói' (fortune telling) and if there is a ghost, we can see it. Then we can do the 'mo' (warding ritual)" *(Spiritualist, Red Dao). He talks in tongues and counts his fingers and says that the disease does not take a comprehensive ritual or large offerings to chase away the disturbing ghost. He chants and burns incense to contact the ghost while offering small gifts of rice, meat, and home-brewed wine. But the child keeps defecating and crying during the evening and night. The mother is now unsure of what she should do since she is *"inexperienced as a mother"*. She has not attended village meetings where health advocacy takes place and there is no women's group in her village and she doesn't understand what is communicated on the radio and TV in Vietnamese. She places her trust in her mother-in-law who decides to prepare a bitter drink of boiled guava buds for the child. During the next five days the mother-in-law attends to the child and feeds it with the drink thrice daily, while the mother is away working in the fields. The child gradually stops defecating liquid stools and regains some strength.

***Using drugs and government health services*** The mother did not go to the CHS for medication, but preferred drugs from the pharmacy in town, which she perceived were of a higher quality. After another six days at home the diarrhoea resumes and the mother now perceives it as a 'serious' *(nang) *disease. She wants to go to the CHS, but: "*I don't know how to ride a motorbike and my husband was not at home"*. The local custom prohibits her to sit on a motorbike with another man, and the CHS is too far away for her to walk to and she feels unable to communicate with any of the health workers at the CHS in Vietnamese. The parents-in-law decide to send her brother-in-law with the sick child to the CHS. He returns with some sachets of powder; a fever reducing drug and ORS, and written instructions on correct dosages of the medicaments in the medical notebook of the child. He was told to ask the VHW for further explanation if needed. The mother is illiterate and says that she never meets the VHW, who lives on the other side of the hill. Further, he never comes around to advice on child health she says. So she decides to give both powders the same way; two - three times a day in a teaspoon of boiled water or tea until the child stops defecating liquid stools. She is very afraid that the diarrhoea would not stop before the medicines run out, she says. But after three days of medication the child recovers and the mother is relieved that she would not have to ask her family-in-laws to take the child back to the clinic or to the district hospital, which would have been very time consuming and costly for the family.

**Table 3:** A lowland case of child diarrhoea management

*Discovering the disease* One evening a 25-year old mother from a Tày lowland village discovers that her 17-month old child is slightly feverish and coughing. Without any objections from her family, she stays at home the next day to attend the child. In the morning the child starts defecating very watery and yellow-greenish stools. The mother describes the child as weak, tired, crying, thirsty, having stomach pains and only wanting breast milk.
***Treatment in the home*** The mother immediately initiates a range of treatments at home: To ward off the fever caused by the *"cold winds hitting the child"*, she performs the traditional *'đánh gió' *(hitting the wind); with a silver coin, an egg and some ginger mixed in a piece of cloth she strokes the head and body of the child. At the same time she gives the child a dose of a fever reducing drug, a left-over from the previous time the child was sick. Maybe the diarrhoea was caused by 'incompatible foods' she says. She therefore restricts the child's diet from such 'harmful foods': *"Avoid sour and fishy things such as eggs and we didn't have any vegetable soup in 3-4 days. I didn't dare to let him eat it!"*. She urges the child to drink water and eat rice mixed with water, salt and minced meat. She also restricts her own diet from green vegetables, being afraid of passing any harmful substances on to the child in the breast milk. On the second day the diarrhoea stops and the mother is happy to have handled this 'light' *(nhe) *diarrhoea at home. Apparently this treatment was 'compatible' to the disease of the child, she says. When asked how she knew what to do, she says that she always asks her mother-in-law and other elder women in the village: "*They know everything. They have been through it all"*. She has also noticed some campaigns during epidemics and some advice sanitation when watching TV at the neighbours. At the regular Women's Union meetings she also learned about child care and personal hygiene. Finally, she prepares a herbal drink for the child. The old people in the village told her to do so: "*The old women said that they brought up many children in the past. They all collected the herbal medicines for drinking"*. She finds the herbal medicines more 'compatible' for a body of a child since they are not harmful as drugs can be she says. She also finds it more practical and effective compared to drugs: "*The clinic medicine is drunk just twice per day. About the herbal medicine, he can drink it anytime he's thirsty. I just let him drink whenever he asks for drinking water. I boil it and leave it in the pot"*. Furthermore, she can drink it herself and pass the healthy substances to the child in the breast milk - unlike any drugs given directly to the child. After four days of this combination of treatments the child recovers and the mother is proud to have successfully handled this disease of her first child.

***Using drugs and government health services*** But after another four days the diarrhoea begins again. This time the mother immediately goes to the CHS on her bike without involving her husband or parents-in-law. She now describes the diarrhoea as serious and therefore not treatable at home. When asked why she did not firstly consult the VHW she says: *"She doesn't have medicine to provide the people. And she doesn't care about the child's diarrhoea"*. At the CHS she does not tell the assisting doctor about the treatment she carried out at home or what might have caused the disease. She returns home with four kinds of drugs, which she knows as: antibiotics, fever reducing drugs, digestion enzymes and ORS. Without consulting anybody, she decides to only give the fever reducing drugs and the enzymes. She finds the antibiotics 'incompatible' with a sick child's stomach. It is *'too sweet' *and *'too hard' *for the child which can cause even more diarrhoea. She does not know what the ORS is for and did not dare ask the CHS staff: *"They are often cross with me. They think I come too often to ask for medicines*", she says.

The two cases show that the non-professional 'popular medical sector' constituted by health care taking place within the spheres of family and community [27], plays a crucial role in the treatment of sick children among ethnic minorities of northern Vietnam. It is within this popular sector that all treatment is initiated and where families negotiate whether to contact the professional sector, such as government health facilities, or the 'folk sector' with other health specialists, such as spiritualists or herbal experts [27]. Secondly, the cases depict several of the social, economical and structural obstacles identified for using the free government health services. These obstacles are investigated below.

### Economic and logistical constraints to accessing health services

Some clear differences in treatment seeking and health care choices were found between the poorer ethnic groups living and working in the highland (Dao and Xa Phó) compared with richer groups living in the lowland setting (Tày and Giáy). As described in case 1 (Table 2), greater difficulties in assessing treatment at health facilities were observed for highland caregivers living furthest away from CHSs and hospitals, compared with the richer and more centrally located communities in the lowland.

Even though health insurance would cover the direct costs of treatment and admissions, highland caregivers (e.g. case 1, Table 2) in particular worried a lot about the substantial indirect costs such as transport, buying food, arranging home-sitting, and paying for extra drugs and services at the CHS or hospitals. Distance and time was also a constraint for highland caregivers from poor families without means of transportation, who would have to spend half or a full day if walking to the CHS. Caregivers acknowledged that this was too time consuming if a child was seriously sick. In focus group discussion and interviews, caregivers all said that they would borrow or spend their money to pay for transport to get to the CHS in time, despite having very low incomes. The cost of petrol or renting a motorbike driver for a round-trip from a highland village and the CHS was 20-25,000 Vietnamese Dong, equalling several days of work for a highland farmer. In comparison, lowland caregivers (Giáy and Tày) lived a maximum of five kilometres away from the CHS and all had bicycles or motorbikes accessible within their households.

### Gender roles as a constraint for accessing health services

Observations in households (conducted during fieldwork in 2008) and interviews with caregivers confirmed that all four groups of ethnic minorities live according to patriarchal gender roles, with the oldest men taking all major decisions and the youngest women being the main domestic labour force. This was most strongly expressed in highland families, where young mothers were also the main agricultural labour force of the families, and who expressed low decision taking powers about their children's health, which complicated seeking the government health facilities (Table 2). In the highland, six out of 19 caregivers of children during sickness were grandmothers, and mothers and grandmothers indicated that elders were the primary persons in the household in charge of deciding, preparing and administering the treatment for a sick child. Mothers were not allowed to travel unaccompanied or with male non-family members to health facilities and needed permission from elders to stay at home from field work to care for the sick child (Table 2). A group of highland men explained: "*when the child gets fever, falls ill, belly pain or something similar, the grandparents have to take care of the child and prepare the medicines*". The men continued: "*Everything must be discussed with the grandparents [...]. After discussing, if all agree, we let the child go (to the CHS)" *(FGD, Dao men).

Highland women also had very few opportunities of accessing information in the community on health care options and preventive measures compared with women in the lowland. TVs and radios were only accessible in few households and not broadcasted in local languages, making it difficult for most women to understand the provided information since they had short or no education and therefore did not speak much Kinh. Village meetings were mostly attended by male heads of households and women's groups were not established in the highland communities. Furthermore, VHWs were not consulted much (elaborated later). In comparison, many lowland family units were smaller and less influenced by the older generations. A group of Xa Phó fathers said: "*The men here all live separately (from their parents). They do not depend on the grandparents; if our child is sick, the husband and wife are in-dependent" *(FGD, Xa Phó fathers). Most lowland mothers (Giáy and Tày) also had basic education and spoke fluently Kinh. They all stressed that they took decisions on their own about staying home from work to tend the child, going to the CHSs or buying drugs in town. Most lowland women also attended Women's Union meetings in their communities regularly, watched TV on daily basis, and could understand the radio and loudspeaker announcements broadcasted in Kinh language.

### Reluctance towards government health services

Despite the fact that all caregivers, from highland as well as lowland communities, highly appreciated access to Government health services and drugs free of charge, many caregivers sought these services with some apprehension. None of the interviewed caregivers sought the advice or were visited regularly by the VHWs working in their villages. Caregivers said they normally only had contact with the VHWs when attending child vaccinations or child nutrition surveillance on a monthly or bi-monthly basis, which were also perceived to be the main tasks of a VHW; "*He just weighs the small children once per month. I only saw him doing that"*(Xa Phó mother). As expressed in both cases (Tables 2 and 3), caregivers also said that VHWs did not have important health services to offer sick children such as drugs, first aid, ORS, or good medical knowledge. All caregivers had therefore experienced receiving a standard advice of going to the CHS from the VHW. This made them go directly to the CHS without consulting the VHW first.

Health Station staff, on the other hand, were perceived to be qualified to diagnose diseases and prescribe drugs. But as exemplified in case 2 (Table 3), the majority of caregivers had bad experiences when consulting the CHS. Their illiteracy and 'backward' lifestyles were often commented on negatively by different types of health staff. Thirteen caregivers told stories of being scolded, ignored, or blamed for seeking treatment too late or too often. A caregiver from the highland explained; "*Most of the people here are scolded by the doctors. Doctors often blame people for not taking good care of their children [...] They should try to understand the situation here. It is really hard (to live) in a place like this" (Dao woman)*. A young lowland mother added: "*I just want the doctors to be more enthusiastic with patients [...] Only when people fall sick, they come to the clinic, so the doctors have to behave softly with them so people can be satisfied" *(Tày mother). Language barriers were also mentioned by caregivers during observations and in all interviews with highland women. They felt they could not communicate accurately in Kinh language with staff at the CHS. Health staff also expressed frustration about not being able to communicate with patients. They therefore used very simple language or, in rare cases, used other waiting patients as translators or asked patients to have someone in their own community read and explain the prescriptions to them (from a prescription book).

To avoid being misunderstood or perceived as backwards, caregivers uniformly said that they never shared ideas about causes of diseases, asked clarifying questions about the prescribed drugs or told any health staff including VHWs, about the home-made treatments they had used before coming to the CHS. This was verified during observations at the CHS. Here, no caregivers were observed to share any information on the child disease or ask about prescriptions, diagnose, or the guidelines given by health staff. Staffs were observed to only ask few direct questions on disease symptoms and personal details.

### Explanatory model for treatment seeking: 'Praying in four directions' and perceived severity of disease

As illustrated in the two cases (Table 2 and 3), caregivers sought and chose treatment according to a pragmatic logic of medical pluralism, applying and combining a range of treatments from different medical systems to increase chances of effect and recovery. This strategy was described with the Vietnamese proverb 'praying in four directions' (*Vái tứ Phương*): *"We call it 'praying in four directions'; when we get sick; we do whatever we have to" (FGD, men, Highland)*. A highland mother added: *"It is safer when doing all. Because sometimes doing 'mo' will (make him) recover, sometimes going to ask for drugs at the health clinic will" (highland mother)*.

As described in the two cases (Table 2 and 3), caregivers commonly shifted between folk, popular, and professional bio-medical treatment systems as a disease developed and choices were often based on the perceived severity of the disease. Home-administered remedies, including herbal mixtures, concoctions and massage were perceived sufficient for 'light' or 'minor' *(nhẹ) *episodes lasting two - four days with irregular defecation of liquid stool, belly pains and general uneasiness. This type of diarrhoea was also described as a 'normal disease' (*bệnh bình thường*) common during childhood, easy to treat and not severely affecting the child: *"The old people said that the children have diarrhoea when they grow up [...] It's not a special thing if a child gets diarrhoea" (Xa Phó mother, lowland)*. A lowland mother added: *"I saw the diarrhoea as normal; he had diarrhoea for three-four days and then recovered. [...] He didn't lie down and he went out to play all day as usual" (*Tày mother). Among all caregivers, diarrhoea was perceived 'heavy' or 'serious' *(nặng) *when lasting more than 2-4 days, re-emerged shortly after recovery or included high fevers, sudden onset of very frequent defecation, and the child becoming very lethargic, or losing weight. In these cases all caregivers mentioned consulting the CHS and taking 'western medicines' *(thuốc tây) *as a necessary treatment. Referral to a hospital was also generally agreed as a necessary action if treatment with western drugs did not stop the diarrhoea. This was also acknowledged among older caregivers from highland communities with longer distances to the CHS; *"When he kept being very sick, we couldn't let him stay at home. We went down to the health clinic to ask for drugs... He was sick at home for two-three days and it was getting too serious... The longer we stay at home, the more serious it gets" *(Dao Grandparents). Nevertheless, a total of six children (four from the highland) had been sick with diarrhoea for several weeks and were seriously weakened before being taken to the CHS and admitted up to 12 days at the inter-communal clinic or the district hospitals receiving frequent intravenous sodium glucose solution and drugs.

### Explanatory model for treatment seeking; 'Compatibility' as a guiding principle

Respondents frequently used the expression of a treatment or method being 'compatible' *(hợp) *or 'suitable' *(phù hợp) *for a child with diarrhoea. Compatibility turned out to be a central and complex concept, functioning at various levels and having implications on all aspects of diarrhoea management, including the perceived cause, the chosen treatment and evaluations of its outcome.

#### Compatibility and causal factors

Reflections about the possible causes of a diarrhoeal episode were important for the choice of treatment. In all four groups of EMGs, a local cosmology of keeping the body of mother and child well-balanced and un-exposed to certain 'un-suitable' elements in the environments or 'in-compatible foods and drugs', dominated the explanations of diarrhoea. 'Unsuitable' environmental factors for children included exposure to sudden changes in weather e.g. from hot to cold or rainy weather or being exposed to something dirty, e.g. dirty playing environment or eating dirty foods. 'Un-suitable' items for children's stomachs' causing diarrhoea mainly included too hot, sour or sweet items, including sweet drugs, foods with 'fishy' *(tanh) *taste or smell (e.g. eggs), sour vegetables or fruits, fatty foods and 'hot breast milk' (from a mother who had worked in the sun). As described in case 2, the suitable treatment for exposure to these factors was to re-balance the body by restricting the behaviour (not going out in the sun etc.) or diet of the child or the breastfeeding mother (Table 3). In all four EMGs, a diet for a sick child often included rehydration measures such as giving rice porridge *(cháo) *with salt and sugar, green teas or herbal concoctions. All breastfeeding mothers also said they had continued breastfeeding during diarrhoea unless they were forced to go to work.

#### Compatibility and treatment testing

The process of testing and establishing the 'compatibility' of a treatment was crucial for disease management. In general, caregivers perceived a treatment to be compatible, if they saw the diarrhoea lessening or stopping within a short time. As in case 1 (Table 2), this was expressed clearly by highland caregivers who always sought spiritual treatment before or parallel with CHS based treatment. This was perceived a compatible treatment for children affected by angry ghosts, a spell or a discontent ancestor, since such factors had to be eliminated first in order to make the child susceptible to biomedical treatment: "*The CHS checks if the child is having a disease. [...] But at home the 'thầy mo' (spiritualist) keeps doing 'bói' and 'mo' (fortune telling and warding) to make the child recover or not. When finishing 'bói' and water transmissions (intravenous sodium glucose solutions) and injections we can see if the child can recover" (Dao men, FGD)*.

#### Compatibility and choice of medication

All types of medication were also evaluated according to whether it was perceived compatible with the specific person or the specific disease. A lowland father explained the choice of herbal medicines: *"If the child is compatible (hợp) to that herbal plant, he/she will recover soon [...]. If that medicine was not compatible, then we go to the health clinic"(Giáy father)*. A lowland mother similarly explained the effect of western medicines: *"Depending on which medicine is compatible to him, he will recover after drinking it 1-2 times" *(Giáy mother). A group of highland fathers explained how they also had to consider the compatibility of a disease with a drug:*" If the disease is suitable to the foreign medicine, you take a little bit to recover [...] (FGD, Dao fathers)*. As already mentioned, shifts from herbal medicines to western drugs were related to perceptions of severity of disease.

As the quote indicates and as described in case 2 (Table 3), these perceptions were linked to general perceptions of 'western drugs' being very powerful and therefore potentially harmful and 'incompatible with the body' compared with natural medicines. Antibiotics were perceived as particularly powerful. Six caregivers described them as 'too hard', 'too strong', and 'too sweet' for a sick child. Unfortunately, as exemplified in case 2 (Table 3) and confirmed in interviews and by observations, it was common to receive 2-4 different kinds of prescribed drugs for diarrhoea at the CHS, with antibiotics and fever reducing powders the most frequently mentioned. In order to limit the intake and harm from western drugs, many caregivers said they gave smaller daily doses of the drugs than prescribed. Or they decided to give only some of the drugs and shifted from one drug to another if recovery did not take place quickly.

These perceptions also had implications for the use of ORS. Only one Xa Phó mother knew how ORS differed from drugs. Others perceived it as any other drug, did not understand the principle of rehydration and did not believe that the child could tolerate the large quantities of powder mixed with water or the unpleasant taste of it. A highland mother explained: *"They (CHS staff) said: mix it with a bowl of water but the child couldn't drink it all. I had to mix with a little bit of water for him to drink it. Then it was sweet and the child liked to drink. If the water is too acrid (chát), the child doesn't like to drink"(Dao mother)*.

Some caregivers also expressed suspicion and doubts about the quality and effectiveness of the free western drugs handed out at the CHS:: "*In the health clinic they are afraid that we do not know how to drink it. They give us the diluted (loãng) medicine"(Highland father)*. Therefore, despite having very low incomes or living in remote areas, some caregivers occasionally bought drugs at private pharmacies in town instead expecting higher quality drugs: "*When we buy, we pay the money and they will give us a good drug so we can get well soon"(Highland mother)*. Hence, the expectations to the effectiveness of western medicines and the easy access to drugs provided by commercial providers seemed to sometimes override concerns of cost, distance and harmfulness of drugs.

#### Summing up

Findings suggest that logistical and social barriers exist for accessing the CHSs. In addition, two guiding principles constitute a local explanatory model for managing childhood diarrhoea among ethnic minority caregivers in this area. Simultaneous resort to multiple treatments or "*praying in all four directions" *is the leading strategy, relying on the two core concepts of 'severity' and 'compatibility'. Reflections of compatibility were drawing on local cosmologies of balancing the body, as well as testing and determining effectiveness of various therapies and drugs.

## Discussion

This study describes how logistic constraints, gender roles, local treatment seeking strategies and reluctance towards government health workers together constitute a suboptimal use of free government health services among ethnic minority caregivers in two rural communes in northern rural Vietnam. These constraints are discussed in the following to suggest ways of improving the quality and use of government health services.

### Economic and logistical constraints when choosing health provider

Low usage of government health providers among EMGs compared to Kinh have previously been ascribed to failures in insurance coverage [19] and logistical constraints to access health services from the highlands [19,28]. But as pointed out by London [8], health service fee exemptions only cover one component of health service costs, while indirect costs may present bigger challenges for the poor. This was also expressed by highland caregivers in this study, who worried a lot about the substantial related costs of admittance, which potentially delayed seeking treatment for seriously sick children.

Interestingly, despite having insurance with access to free treatment for their children at government CHSs, higher costs and longer distances, this and one other study [19] have identified a willingness among ethnic minority caregivers to buy drugs from private drug stores. The common practice among the rural population of Vietnam of seeking private health services and buying over-the counter drugs for self-medication [5,7] apparently extend to poor EMGs.

The study also showed that VHWs, who are the closest health providers, were not considered qualified by caregivers, who instead bypassed VHWs and travelled long distances to access private or public health facilities. Problems of distance to health services might therefore be lessened by upgrading services at community level, either by increasing skills and competences of VHWs in remote communities to perform effective health promotion and basic treatment, or by increasing the frequency of outreach visits by CHS staff to remote communities.

### Balancing between a local explanatory model of disease management and government health services

The study also identified a local explanatory model of disease management among ethnic minority caregivers, clearly differing from a bio-medical treatment system. Simultaneous resort, local medical cosmologies of obtaining bodily balances, concepts of compatibility of treatment and humoral qualities of medicines (e.g. hot/cold and heavy/light) as identified in this study are all commonly found in the Asian context of medicine and well described in minority as well as majority populations in Asia and Vietnam [7,29-31]. However, our study also found that health staff ascribed such health seeking patterns as 'ethnic' and 'backwards', and that government health services are not always the first and most well-liked choice of health service for ethnic minority caregivers.

Health is a key objective of state policies and governance in Vietnam and health sciences in modern Vietnam have become symbolically associated with socialist modernity, rationality, and progress [8,32]. Further, ethnic minorities have commonly been described as 'marginal' and 'developmental backwards' and therefore targeted by the socialist state to 'assimilate' to mainstream developmental standards [33,34] - while also being encouraged by the state to preserve cultural traditions not posing any threats to progress of the socialist state [33,35]. 'Superstitious beliefs and practices' such as shamanistic rituals and animal sacrifice have been deemed as 'backward' health practices and a constraint for progress and modernity [32,35]. Hence our study provide further evidence that ethnic minorities seem to balance between practicing, to them, meaningful health rituals, while also interacting with a modern government health system. This might explain why ethnic minority caregivers do not reveal local explanatory models of disease to health staff and opt for drugs from the private sector, where they are not met with demands to change health behaviours.

### Improving health services and health staff competencies for ethnic minorities

This study agrees with previous findings on health service use in Vietnam, that low trust and dissatisfaction with medical staff [17], and discouraging communication with health personnel affects disadvantaged women's and ethnic minorities' treatment seeking behaviours and use of the public health sector [28,36]. This is underlined in a recent World Bank report stressing that female ethnic minority caregivers in Vietnam are particular vulnerable and in special need of appropriate health services [34]. The public health system of Vietnam has been described as particularly 'provider-centred', not anticipating patients to speak up their needs or questioning health providers [37,38]. This is possibly worsened for illiterate EMGs who also live in areas with poor health service coverage and where it is difficult to attract and retain qualified health staff [2]. Hence, this study argues for a need for the Vietnamese health system to focus on service levels in remote areas of the country and to change the approach to marginal population groups in Vietnam to increase their trust in and use of government health services.

Cross-cultural competence models [39,40] have been developed to address communication challenges in the health sector. They stress that health staff working in ethnically diverse populations must understand patients' actions and perceptions of disease as expressions of their social realities, including constraining socio-economic factors and local explanatory models of disease. For health staff in daily contact with EMGs in Vietnam this clearly implies acknowledging the flexible 'praying in four directions' approach to choosing treatment and local cosmologies of disease management, as well as avoiding the persisting stereotyping of EMGs as 'culturally backwards'. To address these needs, Vietnamese Medical Universities have recently strengthened community health training of medical students [41], while other staff groups remain to be considered. This study primarily focused on understanding the perspective of ethnic minority caregivers' on use of child health services. Further studies into the constraints of delivering effective government health care in mountainous areas of the country, would add important information to understanding the full complexity of providing effective health care for all children in Vietnam.

Our findings also corroborate other studies highlighting the potential problem of self-medication and over-prescription of drugs to children in Vietnam. Training of health staff should therefore focus on improved prescription practices and guidance to patients on how to administer drugs and ORS. They should encourage open discussions with patients on when to expect effects from drugs, and when to shift treatment and health care system. Small-scale interventions have managed to improve drug prescription and purchasing practices in urban sites in Vietnam [42-44]. It is strongly recommended that such interventions be scaled up to include rural and mountainous areas, where ethnic minorities are in need of good guidance.

### Opportunities for improved health promotion and trust in government health systems

Empowering and encouraging Vietnamese caregivers with good care practices can enhance and sustain better child-care and health care practices in rural communities [45-47]. The current study highlights that most caregivers from all four EMGs recognize symptoms and evaluate the severity of diarrhoea within few days of onset and respond caringly and rapidly by applying various treatments. However, caregivers felt that hardly any encouragements or preventive advice were given to support these care giving efforts during contact with VHWs or CHS staffs. We highly recommend that all staff at government health facilities use every opportunity to praise and encourage such good care giving behaviours. This can form a fruitful starting point for further integrating preventive messages and child health promotion into the contact with caregivers. This may strengthen caregivers' experience of being in contact with a caring and trustworthy health system.

In the present study we also saw strong indications of patriarchal gender roles creating difficulties for young highland mothers in particular to follow health messages and seek CHSs, depending on male and older family members' permissions. These findings are in agreement with other studies in the rural population of Vietnam [30,48], and other ethnic minorities in Asia [29], which show that older community members and grandmothers, in particular, play core roles in child disease management and care. However, current child health promotion programs in Vietnam and health promoters mainly target and educate mothers as sole caregivers. It is therefore strongly recommended that health promotion in Vietnam include other family members as key people in children's 'care groups' and their resources in child care. These people can be reached through different grassroots organisations (e.g. Farmers' Unions and Women's Union) or community stakeholders in daily contact with men and elders, e.g. village heads, diviners and CHS staff.

Finally, we acknowledge the beneficial practices of rehydrating children with diarrhoea using continued breastfeeding and home-made oral rehydration solutions of green teas, water or rice gruels with added salt and meats. These practices should be known to all health providers including spiritualists, pharmacists and VHWs as beneficial and included as advice during contact with caregivers. This might be more effective than prescribing ORS to less severe cases, since it is perceived 'compatible' with children, while ORS is perceived a non-compatible western drug and also administered incorrectly by many caregivers.

### Study strengths and limitations

This study has provided new information on the qualitative aspects of why and how ethnic minority caregivers in Vietnam use and choose health services for child diarrhoea. However, despite triangulation of methods and a thorough sampling strategy the study faces some limitations:

Firstly, as also pointed out by Toan et al [19], the geographical inequity in access and use of government and private health services, as well as language barriers and poor economical conditions, are anticipated to be stronger for EMGs living in less developed areas of the country e.g. in high mountains with longer distances to health facilities and urban environments. Secondly, difficulties of gaining access to 'the insider perspective' as outsiders to a research community can present serious limitations to qualitative research [49]. Since some caregivers expressed being marginalised and disrespected by Kinh people due to their ethnicity, the researchers' and assistants' backgrounds (a white westerner and of Kinh origin from urban developed environments) might have caused reluctance from respondents to disclose actions which could be perceived as 'wrong', 'traditional' or 'backward'. This problem was anticipated by the first author having good prior knowledge of the living conditions and having socialized with many community members and their children during a previous six month long fieldwork in the same study area. Being outsiders to the area was also mentioned as a benefit by some respondents, who felt safe to disclose bad experiences with health staffs and family members since the researcher and assistants were not affiliated with any local authorities or organisations. However, it did constitute a special challenge to gain the trust of young highland mothers living in patriarchal families and making them speak up about sensitive issues of gender and decision making. Research assistants therefore aimed at interviewing mothers without the presence of elders and men and encouraged trusted friends and sisters to take part. Thirdly, some information is likely to be lost or transformed during translations with the first author not speaking Kinh, the research assistants not speaking ethnic minority languages and the need for double translation in Xa Phó and Dao communities where some women did not speak Kinh. The quality of translation was validated as research assistant's cross-checked and discussed recordings and each other's transcriptions of all interviews and FGDs with the first author.

## Conclusions

This study investigated treatment seeking strategies for child diarrhoea among four groups of ethnic minority caregivers in northern Vietnam. The study identified several socio-economic and logistical constraints for highland caregivers in particular to seek treatment, while also identifying a local explanatory model which guided disease management among caregivers in all four ethnic groups. Suggestions for future child health promotion include increased quality of government health services, including improved communication skills and drug prescription practices of government health staff. Village health workers who are important primary health providers, but not sought by caregivers, should also be upgraded. Broader health promotion programs should address the strong patriarchal gender norms limiting highland mothers to seek treatments. Encouraging existing good child care practices and including elder family members as important caregivers, also present important opportunities for effective future child health promotion.

## Competing interests

The authors declare that they have no competing interests.

## Authors' contributions

TR was responsible for and carried out the data collection, analysed the data material and drafted the manuscript. FK and HS participated in designing the study and FK, HS, and AD provided comments on the analysis and revised the manuscript together with TR. All authors read and approved the final manuscript.

## Pre-publication history

The pre-publication history for this paper can be accessed here:

http://www.biomedcentral.com/1471-2458/11/690/prepub

## Supplementary Material

Additional file 1 **Caregiver Interview Guide**. The interview guide which was used during the collection of the main data source; 43 semi-structured in-depth interviews with caregivers of pre-school children (six years and below) who had experienced a case of diarrhoea in the past month. Click here for file
