# Supplementary material for: Perspectives on child diarrhoea management and health service use among ethnic minority caregivers in Vietnam
Source: BMC Public Health. 2011 Sep 6;11:690. doi: 10.1186/1471-2458-11-690 (PMC3189136; doi:10.1186/1471-2458-11-690)
Supplement: Additional file 1 — Caregiver Interview Guide. The interview guide which was used during the collection of the main data source; 43 semi-structured in-depth interviews with caregivers of pre-school children (six years and below) who had experienced a case of diarrhoea in the past month. [file 1471-2458-11-690-S1.DOC]

# Caregiver Interview Guide

**Topic 1. Frequency and description of the diarrhoea**

- How often does your child get diarrhoea?
- When does it normally happen?

**If you think about the last time your child had diarrhoea…**

- What were the signs of diarrhoea that you saw in your child?
- How can you describe the diarrhoea?
- How many times did the children defecate in a day?
- How did the faeces look?

**Topic 2. Perceived causes of diarrhoea**

- What is normally causing diarrhoea in children here in this village?
- Why did your child get diarrhoea you think?
- Can you think of any causes of diarrhoea, which are related to water and sanitation, or personal or public hygiene? (describe)

**Topic 3. Treatment at home**

- What did you do as the first thing when your child developed diarrhoea?

- Then what did you do?

- What do you do if the first thing does not work?

- Do you normally give a child with diarrhoea any kind of treatment? Which kind?

- Did you give the child any special kinds of foods, drinks, any medicinal leaves, or any other treatment at home? (describe)

- Who takes care of the sick child at home?

- Who decides to give treatment to the child at home?

**Topic 4. Sources of child care knowledge and advice**

- Who taught you about the different types of treatment for sick children?
- Where did you get advice on how to care for your sick child?
- If you need an advice about diarrhoea, who do you normally ask?
- Can anyone in the family help you? (who and how?)
- Can anyone in the village help you? (who and how?)

**Topic 5. Going to the health clinic**

- Did you go to the health clinic to get the diarrhoea treated?

- How long time did treat the child at home before going to the health clinic with the child?

- How do you know if you have to go to the health clinic?

- How did you and your family decide to take the child to the clinic?

- Who went with the child to the clinic? (why?)

- How did you/they get there?

- Can you describe to me what happened at the clinic from when you arrived to when you left?

- What did the doctor say to you about the child and the diarrhoea?

- Did the doctor say anything on what to do at home to prevent the diarrhoea to come back?

- What do you think about the staff and the services at the clinic?

- What do you think about the drugs you can get for your child at the clinic?

- What do you think about the drugs you can get other places?

**Topic 6. Preventing diarrhoea**

- What do you do to prevent that your child gets diarrhoea again?

- Do you or your family do anything at your house or in your village to prevent children from getting diarrhoea? (describe)
